# The impact of a water promotion and access intervention on elementary school students in the presence of food insecurity

**DOI:** 10.1017/S1368980024002283

**Published:** 2024-11-22

**Authors:** Leslie Gerstenfeld, Lauren Blacker, Charles E McCulloch, Lorrene D Ritchie, Valeria M Ordonez, Laura Schmidt, Anisha I Patel

**Affiliations:** 1 Philip R. Lee Institute for Health Policy Studies, University of California, San Francisco, San Francisco, CA, USA; 2 RUSH University Medical College, Chicago, IL, USA; 3 Department of Epidemiology and Biostatistics, University of California, San Francisco School of Medicine, San Francisco, CA, USA; 4 Nutrition Policy Institute, University of California, Division of Agriculture and Natural Resources, Oakland, CA, USA; 5 Stanford Department of Pediatrics, Palo Alto, CA, USA; 6 Department of Humanities and Social Sciences, University of California, San Francisco, San Francisco, CA, USA

**Keywords:** Food insecurity, Drinking water, Obesity, Diet

## Abstract

**Objective:**

School-based interventions encouraging children to replace sugar-sweetened beverages with water show promise for reducing child overweight. However, students with child food insecurity (CFI) may not respond to nutrition interventions like children who are food-secure.

**Design:**

The Water First cluster-randomised trial found that school water access and promotion prevented child overweight and increased water intake. This secondary analysis used mixed-effects regression to evaluate the interaction between the Water First intervention and food insecurity, measured using the Child Food Security Assessment, on child weight status (anthropometric measurements) and dietary intake (student 24-h recalls, beverage intake surveys).

**Setting:**

Eighteen elementary schools (serving ≥ 50 % children from low-income households), in which drinking water had not been previously promoted, in the San Francisco Bay Area.

**Participants:**

Students in fourth-grade classes (*n* 1056).

**Results:**

Food insecurity interacted with the intervention. Among students with no CFI, the intervention group had a lower prevalence of obesity from baseline to 7 months (–0·04, CI –0·08, 0·01) compared with no CFI controls (0·01, CI –0·01, 0·04) (*P* = 0·04). Among students with high CFI, the intervention group had a pronounced increase in the volume of water consumed between baseline and 7 months (86·2 %, CI 21·7, 185·0 %) compared with high CFI controls (–13·6 %, CI –45·3, 36·6 %) (*P* = 0·02).

**Conclusions:**

Addressing food insecurity in the design of water promotion interventions may enhance the benefit to children, reducing the prevalence of obesity.

Childhood overweight and obesity are associated with an increased risk of diabetes, CVD, adult overweight and consumption of ultra-processed foods including sugar-sweetened beverages (SSB)^(1–3)^. Drinking water during meals has been shown to reduce hunger and promote satiety but may not impact calories consumed^(4,5)^. Substitution of water for SSB has been associated with reduced energy intake, increased energy expenditure and increased fat oxidation in studies of obese adults and children^(6,7)^.

Food insecurity, a chronic lack of ‘access to enough food to support an active, healthy life’, is a risk factor for childhood overweight and obesity^(8–13)^. People experiencing an unpredictable food supply may be more prone to weight gain to buffer for times of food scarcity^(14,15)^. The stress of an unreliable food supply may impact self-regulation in the presence of food, decreasing satiety and increasing emotional overeating^(16–18)^. Childhood food insecurity is associated with higher consumption of total calories, fat, sugar and fibre^(11,19)^. Mothers, infants and toddlers with food insecurity are more likely to consume SSB and consume them more frequently than those who were food-secure^(20,21)^. Low-income households are commonly found in areas where there is a high concentration of unhealthy food outlets, many of which sell SSB^(22)^. The relative affordability of SSB, and the ubiquity of SSB, and SSB advertisements in these communities promote the sale of SSB over healthier beverage options^(23)^.

School-based drinking water interventions that promote the substitution of water for SSB have been shown to increase water consumption, reduce SSB consumption, decrease flavoured milk purchases and reduce the prevalence of childhood overweight^(24–26)^. The Water First drinking water access and promotion intervention increased the frequency of water consumption and reduced overweight prevalence among low-income, ethnically diverse, fourth-grade students in the San Francisco Bay Area^(27)^. Studies found that adults with food insecurity experienced reduced benefits from nutrition interventions, but little is known about the impact of food insecurity on children’s responses to nutrition interventions^(28,29)^. Informed by this research, we hypothesised that students experiencing food insecurity would be less likely to benefit from the intervention. This would in turn reduce the impact of the Water First intervention in preventing unhealthy weight gain among students.

## Methods

The Water First cluster-randomised controlled trial was a drinking water promotion and access intervention conducted with predominantly low-income and ethnically diverse fourth-grade students^(30)^. Enrolled schools served low-income households (≥ 50 % of students eligible for free and reduced-priced meals) and were not already promoting drinking water by offering appealing water stations and/or providing cups or reusable water bottles. A total of twenty-six elementary schools (cohorts of 6–8 schools per year) in four school districts in the San Francisco Bay Area, California, were enrolled from August 2016 to March 2020. Half of the schools within each district cohort participated in the intervention, while half served as controls. Data from eight schools enrolled in the 2019–2020 cohort were incomplete due to COVID-related school closures and therefore omitted from this analysis^(27,30)^.

### Intervention

In each intervention school, a tap water dispenser with disposable cups was installed in the cafeteria and two reusable water bottle filling stations were installed in additional high-traffic locations, including areas where students had physical education classes or recess. Students in schools randomised to the intervention were given reusable water bottles and engaged with Water First staff in eight 15-min classroom activities highlighting the health, financial and environmental benefits of drinking water. Schoolwide activities included assemblies and awarding of small prizes to students drinking water. Details of the study protocol are published elsewhere^(30)^.

### Data collection

At three time points, baseline (at the start of the school year), and 7 and 15 months later, Water First staff measured students’ height and weight using methodology consistent with National Health and Nutrition Examination Survey Anthropometry Procedures Manual^(31)^, and students completed surveys reporting frequency of beverage consumption. Diary-assisted 24-h dietary recalls were conducted at baseline and 7 months. Surveys at 15 months captured students’ self-reported child food insecurity (CFI) status^(30)^.

### Outcome variables

The primary outcome for the Water First study was prevalence of overweight (BMI for age and sex: ≥85^th^ percentile). Secondary weight status outcomes included prevalence of obesity (BMI for age and sex: ≥95^th^ percentile), BMI, BMI percentile and BMI *z*-score^(30,32)^. Dietary intake, also a secondary outcome, was assessed in two ways. Diary-assisted 24-h dietary intake recalls conducted by trained researchers using the multiple-pass method were used to evaluate water, food and beverage intake over the previous 24 h^(33)^. Food and beverage calories were estimated using the US Department of Agriculture’s Food and Nutrient Database for Dietary Studies^(34)^. An adapted instrument for students, used in prior studies^(30,35)^, was used to assess the frequency of student intake of plain water, SSB, juice, flavoured milk and plain milk.

### Food insecurity

Food insecurity was quantified using five of the nine statements from the Child Food Security Assessment (CFSA)^(11,36,37)^. The US Department of Agriculture Food Security Survey Module for Youth was not used as it includes questions only on food quality and quantity and is designed only for children 12 years and older^(38)^. In contrast, the CFSA was developed based on interviews with children as young as 7 years old and taps into children’s cognitive, emotional and physical awareness of food insecurity^(36,37,39)^. To achieve a reasonable student survey length, five items from the CFSA were selected as the most accurate for assessing student awareness of food insecurity and reliably measuring food insecurity in children aged 7 years and up^(11,37)^. Students were asked how often in the previous 12 months did they experience the following:

1. We can’t get the food we want because there is not enough money.
2. I worry about how hard it is for my parents to get enough food for us.
3. I worry about not having enough to eat.
4. I feel hungry, because there is not enough to eat.
5. I get really tired, because there is not enough to eat.

In accordance with the assessment guidelines, responses were coded as 0 (never), 1 (1 or 2 times) and 2 (many times) and summed across all statements for a relative CFI score (0–10)^(11)^. The CFI score was categorised into three subgroups: score=0 (no CFI), score=1 or 2 (medium CFI) and score>2 (high CFI)^(40)^. These cut-offs were selected based on distribution to establish categories with similar sample sizes and to provide meaningful interpretation of results. The distribution of CFI scores is presented in Table 1.

**Table 1.** Baseline characteristics of Water First food insecurity study participants

Characteristic	Total (*n* 1056)		Intervention (*n* 547)		Control (*n* 509)		*P*-value
	No./Mean	(%/sd)	No./Mean	(%/sd)	No./Mean	(%/sd)	
Student participants per school, mean (sd)*	58·7	18·8	60·7	16·6	56·6	21·6	NS
Age in years, mean (sd)†	9·6	0·41	9·6	0·38	9·6	0·43	NS
Female, No. (%)‡	500	47·4	255	46·6	245	48·1	NS
Race/ethnicity, No. (%)‡							
Mexican American, Latino, Hispanic	672	63·6	369	67·5	303	59·5	Ref
Asian/Native Hawaiian and Other Pacific Islander	147	13·9	62	11·3	85	16·7	0·005
Non-Hispanic Black/African American	45	4·3	21	3·8	24	4·7	NS
Non-Hispanic White	76	7·2	36	6·6	40	7·9	NS
Other (American Indian/Alaska Native, Multiple)	116	11·0	59	10·8	57	11·2	NS
Physical activity times/week, No. (%)‡							
0 times	105	9.9	59	10·8	46	9·0	NS
1–2 times	406	38·4	213	38.9	193	38·0	Ref
3–4 times	218	20·6	107	19·6	111	21·8	NS
5–6 times	137	13·0	70	12.8	66	13·0	NS
7 or more times	189	17·9	96	17·6	93	18.3	NS
Screen time yesterday in hours, mean (s d)†	3·1	2·9	3·2	2·7	3·0	3·0	NS
Child food insecurity score (0–10), mean (s d)†	1·8	2·1	1·8	2·1	1·8	2·1	NS
Child food insecurity category†							
No food insecurity (score = 0), No. (%)	374	35·4	194	35·5	180	35·4	NS
Medium food insecurity (score 1–2), No. (%)	370	35·0	186	34·0	184	36·1	NS
High food insecurity (score 2–10), No. (%)	312	29·6	167	30·5	145	28·5	NS
Baseline prevalence of overweight/obesity (%)§	518	49·1	272	49·7	246	48·3	NS
Baseline prevalence of obesity (%)||	325	30·8	178	32·5	147	28·9	NS

* Student participants per school assessed using linear regression clustering on school.

† Differences in age, hours of screen time yesterday and food insecurity score by intervention status were assessed using mixed-effects linear regression models accounting for school and class effects.

‡ Percentage of female students, race/ethnicity and frequency of physical activity/week were assessed using mixed-effects logistic regression models accounting for school and class effects.

§ Overweight/obesity is defined as BMI for age and sex: ≥85^th^ percentile.

|| Obesity is defined as BMI for age and sex: ≥95^th^ percentile.

### Covariates

Covariates were prespecified in the Water First study protocol to adjust for potential imbalance that is more common in cluster-randomised controlled trials than trials that randomise at the individual level. Covariates assessed via student self-report at baseline included age, gender, race/ethnicity, physical activity and screen time. Physical activity was assessed using questions from the Physical Activity Questionnaire for Older Children and Adolescents^(41)^. Students were asked how many times in the previous 7 d did they spend their free time doing things that involved physical effort that made them breathe hard or sweat. Reporting categories were never (0 times), sometimes (1–2 times), often (3–4 times), quite often (5–6 times) and very often (7 or more times) in the previous 7 d. Screen time was reported as a continuous variable summed over three categories: playing video or computer games, watching movies or programmes on TV or computer, or doing other things on a computer or phone such as searching the internet, social media, email or texting. Students reported during the previous day how much time they had spent for each category: no time at all, less than an hour, 1–2 h, 2–3 h, 3–4 h, 4–5 h, or 5 or more hours.

### Data analysis

Using Stata version 17, mixed-effects logistic regression models including a three-way interaction between food insecurity, the intervention and time were employed to predict differential changes in weight status, number of times per week different beverages were consumed, and food and beverage energy intake. Models controlled for covariates listed above. Potential clustering of students in classes and schools was addressed in the models through inclusion of random effects for the school, class and student. To achieve convergence of obesity regression models, the covariates were limited to race/ethnicity and potential clustering was addressed through inclusion of random effects for students.

Dietary intake data were log-transformed prior to regression analysis to account for the skew of variable distributions. Regression coefficients were subtracted from baseline estimates for each time period and exponentiated to estimate percent changes in median predicted values for dietary intake outcome variables.

Differences in predicted estimates resulting from interaction of the intervention with food insecurity over time were evaluated for statistical significance. Because of the poor precision in estimating interaction terms, it has been suggested to raise the *P*-value to declare statistical significance of an interaction term to as high as *P* < 0·20^(42)^. To balance multiple testing concerns with this poor precision, we elected to declare interactions with *P* < 0·05 as statistically significant. Outcomes found to be significantly modified by food insecurity and the intervention over time were further evaluated by food insecurity subgroups.

## Results

**Figure 1.**
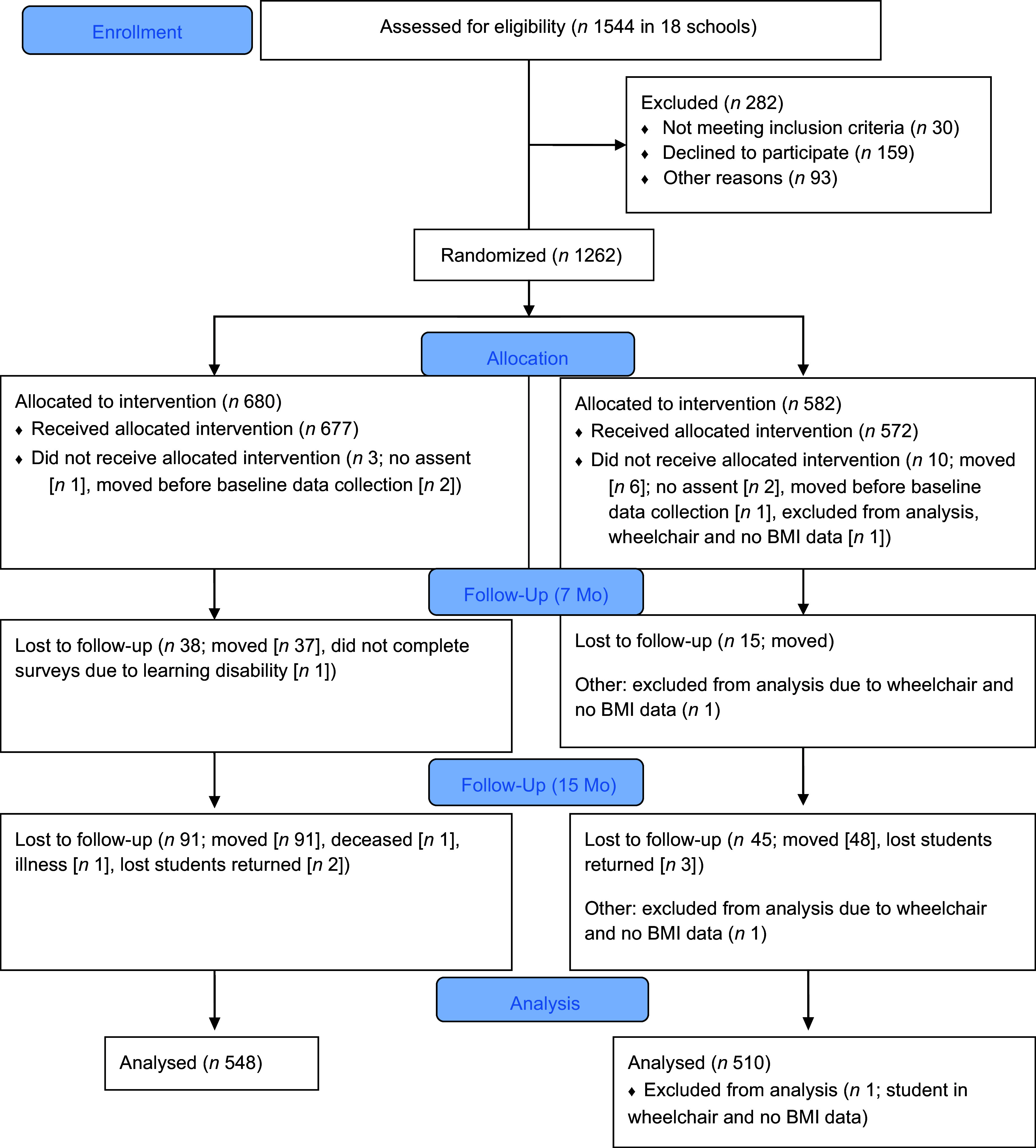
CONSORT flow diagram for the impact of the Water First promotion and access intervention on elementary school students

The study sample included 1056 students for whom food insecurity status was reported (84 % of the total sample – 206 students [16 %] were lost to follow-up or did not respond to the food insecurity questions) (Fig. 1). At baseline, study students had a mean age of 9·6 years (sd = 0·41), 47·4 % were female and 63·6 % were of Mexican American/Latino/Hispanic race/ethnicity. There were significantly fewer Asian/Native Hawaiian and Other Pacific Islanders in the intervention group (*n* 62) compared with the control group (*n* 85) (*P* = 0·005). No other significant differences were found in demographic variables between the intervention and control groups. The food insecurity score distribution was skewed (mean 1·8, sd 2·1) with 65 % of students reporting some level of food insecurity in the previous 12 months (Table 1).

CFI status significantly interacted with the prevalence of obesity (*P* = 0·04) and the volume of water consumed (*P* = 0·04). No significant interaction was observed for the Water First primary outcome, overweight. There were also no significant interactions for other secondary outcomes including: BMI *z*-score, BMI percentile, overall calories, food calories, beverage calories, SSB calories, and frequency of milk, flavoured milk, SSB, 100 % juice and water intake.

Subgroup analyses of significant interactions were conducted to understand the predicted outcomes associated with the prevalence of obesity and the volume of water consumed within each CFI category. Among students with no CFI, those exposed to the intervention had a reduced prevalence of obesity between baseline and 7 months (–0·04, CI –0·08, 0·01) compared with an increase among no CFI controls (0·01, CI –0·01, 0·04) (*P* = 0·04) (Table 2). Among students with high CFI, the intervention group had significant increases in volume of water consumed between baseline and 7 months (86·2 %, CI 21·7, 185·0) compared with a decrease (–13·6 %, CI –45·3, 36·6) observed in the high CFI control group (*P* = 0·02). There was no evidence of significant interaction between the intervention and CFI relative to other outcomes of interest.

**Table 2:** Changes in prevalence of obesity and water intake from pre- to post-intervention by child food insecurity categories

Outcome variable	No food insecurity*					Medium food insecurity*					High food insecurity*				
	Intervention		Control		*P* †	Intervention		Control		*P* †	Intervention		Control		*P* †
	Estimate	95 % CI	Estimate	95 % CI		Estimate	95 % CI	Estimate	95 % CI		Estimate	95 % CI	Estimate	95 % CI	
Weight status															
Prevalence of obesity‡															
Baseline prevalence	0·29	0·22, 0·35	0·22	0·20, 0·24	ref	0·23	0·19, 0·27	0·25	0·20, 0·31	ref	0·24	0·19, 0·28	0·23	0·19, 0·26	ref
Prevalence changes 7 months minus baseline	–0·04	–0·08, 0·01	0·01	–0·01, 0·04	0·04	0·02	–0·02, 0·06	–0·01	–0·05, 0·02	0·19	–0·01	–0·04, 0·02	–0·01	–0·04, 0·02	0·82
Prevalence changes 15 months minus baseline	–0·04	–0·08, 0·01	0·01	–0·01, 0·03	0·06	0·01	–0·02, 0·04	–0·01	–0·05, 0·03	0·52	0·02	–0·01, 0·06	–0·02	–0·04, 0·01	0·08
24-h diary-assisted dietary intake§															
Plain water (fluid ounces)															
Baseline	6·12	4·50, 8·42	7·54	5·44, 10·48	ref	5·68	4·09, 7·91	3·75	2·71, 5·21	ref	3·58	2·54, 5·04	4·57	3·15, 6·63	ref
% Changes 7 months minus baseline	–20·5	–46·5, 18·0	–7·3	–38·3, 39·4	0·60	8·6	–27·9, 63·6	39·3	–6·9, 108·5	0·40	86·2	21·7, 185·0	–13·6	–45·3, 36·6	0·02

* Child food insecurity score based on student survey responses to five questions from the Child Food Security Assessment. Responses were coded as 0 (never), 1 (1 or 2 times) and 2 (many times) and summed across all statements for a relative food insecurity score (0–10)^(11)^. The child food insecurity score was categorised into three categories: score = 0 (no child food insecurity), score = 1 and 2 (medium child food insecurity) and score > 2 (high child food insecurity)^(40)^.

† *P*-value calculated from analysis of regression model for interaction of food insecurity, intervention and time.

‡ Multilevel mixed-effects logistic regression models used to examine intervention impacts on changes in outcomes adjusting for intervention status, time point and race/ethnicity. Models included random effects for students.

§ Multilevel mixed-effects logistic regression models used to examine intervention impacts on changes in outcomes, adjusting for intervention status, time point, age, race/ethnicity, gender, screen time, physical activity and time. Models included random effects for school, class and student change over time.

## Discussion

Consistent with our hypothesis, the Water First intervention did not reduce the prevalence of obesity among children with food insecurity even though it was effective for others^(27)^. The change in the prevalence of obesity was significantly lower among students with no CFI in the intervention group (–13·8 %) compared with the control group (4·5 %). There was no significant difference between the change in the prevalence of obesity over time among students with CFI in the intervention group compared with those in the control group, suggesting that students with no CFI may have benefitted more from the intervention.

Among students with high CFI, those in the intervention group significantly increased their volume of water intake during the trial. Increased water intake is likely to be attributable to the intervention, which focused on water promotion. Concomitant reductions in SSB consumption were not observed in students with CFI exposed to the intervention. This study was not equipped to investigate the intervention’s mechanisms of action, but the literature suggests a range of possible mechanisms. Prior studies find that adults and children with food insecurity frequently eat beyond satiety and experience emotional overeating^(18)^. Moreover, the SSB industry selectively price and market their products to low-income consumers^(23)^, and SSB intake may be habitual in households with food insecurity as a low-cost source of calories^(21)^. High-calorie, low-nutrient diets have been identified as a potential link between food insecurity and poor health outcomes that may be impacted by interventions^(20,21)^.

As a result of these possible mechanisms, children with food insecurity could experience higher barriers to drinking water over SSB that were not overcome by the Water First intervention. Results suggest that water promotion efforts should be designed in ways that enhance the benefit to children with food insecurity. Like other studies^(24–26)^, Water First focused on making changes to the school environment and did not evaluate or impact the availability of water outside of school. Nor did it alter the widespread availability of ultra-processed foods, including SSB, in the child’s food environment at home or in the community^(43)^. Future studies should attempt to maximise the benefit of water promotion by engaging parents in the intervention with the intent of impacting the food environment both inside and outside school, especially in low-income communities.

A strength of this study is the use of student self-reporting to define food insecurity. Self-reporting has been identified as a more accurate assessment of CFI compared with parent reporting. Even when parents reported shielding children from household food insecurity, children reported household food insecurity^(36,44)^.

This study has several limitations. The control group included significantly more Asian/Native American and other Pacific Islanders than the intervention group; analyses controlled for within person changes so any potential bias should be minimal. Estimation of interaction terms coupled with multiple testing issues introduce poor precision to regression models used in this evaluation; selecting a significance level of *P* < 0·05 is conservative, but these precision concerns may limit the relevance of the results presented in this study. The Water First cluster-randomised controlled trial was not powered to evaluate subgroup interaction effects on the intervention. CFI was only measured at the 15-month follow-up and therefore may not accurately reflect changes in the level of food insecurity in the study population throughout the study period.

## Conclusion

School-based drinking water interventions may be impacted by the presence of CFI among students. Future, adequately powered studies may enhance the understanding of the interaction between nutrition interventions and food insecurity. Consideration of food insecurity in the design of nutrition interventions may maximise the benefits to all populations.
